# The Influence of Fiber Tension and Filament Winding Patterns on the Strength of Thin-Walled Fiber-Reinforced Polymer Composite Tubes

**DOI:** 10.3390/polym18111394

**Published:** 2026-06-04

**Authors:** Karolina Paczkowska, Zuzanna Pacholec, Wojciech Błażejewski

**Affiliations:** Department of Mechanics, Materials and Biomedical Engineering, Wroclaw University of Science and Technology, Smoluchowskiego 25, 50-370 Wroclaw, Poland; karolina.paczkowska@pwr.edu.pl (K.P.); wojciech.blazejewski@pwr.edu.pl (W.B.)

**Keywords:** composite tubes, filament winding, winding tension, winding pattern, DIC

## Abstract

This study investigates the effects of filament winding parameters (tension and mosaic pattern) on the mechanical performance of thin-walled fiber-reinforced polymer composite tubes under internal pressure. The pressure was generated through axial compression of an elastomeric insert, providing a controlled alternative to conventional hydrostatic burst testing. Tubes were manufactured with different combinations of winding tension (10–50 N) in the ±55° and hoop layers. Within the ±55° layer, several mosaic pattern configurations were tested. Structural responses were evaluated using pressure testing, Digital Image Correlation (DIC), and Scanning Electron Microscopy (SEM). 20 N was identified as the most efficient tension level, improving interlaminar integrity and increasing hoop tensile strength by approximately 8–13%. Specimens with a hoop layer failed abruptly by hoop-dominated brittle fracture, characterized by longitudinal splitting and fiber rupture in the circumferential direction. Among the investigated mosaic configurations, the 3/3 pattern demonstrated the most efficient structural response—the mean hoop tensile strength (1088 ± 43 MPa) was approximately 31–40% higher than that of the remaining configurations (722–798 MPa). Overall, the results indicate that both winding tension and mosaic pattern influence the failure pressure, with optimized configurations contributing to improved pressure resistance and structural consistency.

## 1. Introduction

Fiber-reinforced polymer (FRP) composite pressure vessels and pipes are increasingly used in applications requiring high strength-to-weight ratios, such as aerospace, automotive, and energy storage systems. These components are commonly manufactured using processes like filament winding, where the fiber structure and manufacturing process parameters, including fiber tension or resin impregnation quality, highly influence their mechanical performance under internal pressure [1,2,3,4,5]. The growing demand for lightweight materials, coupled with the recent adoption of sustainable manufacturing approaches, further drives the optimization of key process parameters to minimize material usage while maintaining the required strength [6,7]. This balance can be effectively achieved by adjusting parameters such as fiber tension and mosaic winding patterns.

Previous studies have shown that variations in fiber tension can significantly affect the stress distribution, failure modes, and overall strength of composite pressure components [8,9]. Maintaining proper tension is crucial for preserving fiber alignment, ensuring that the fibers follow the intended winding paths without slippage or deviation [10]. Additionally, fiber tension affects the fiber volume fraction: higher tension results in tighter compaction of layers, which reduces resin content as excess resin is squeezed out. This change directly impacts the composite’s stiffness and strength, given that fibers serve as the primary reinforcement. Conversely, too low fiber tension can cause fibers to slip from their intended paths, resulting in loose winding, waviness within the layers, and uneven resin distribution [11,12]. However, excessive tension is also detrimental, potentially leading to fiber breakage, mandrel deformation, increased residual stresses, and fiber misalignment due to fibers being pulled away from their planned trajectories [13,14]. In a previous study [15], Błachut et al. investigated two extreme fiber tensions, 3 N and 80 N, and found that higher tension resulted in increased fiber content and enhanced mechanical strength of the FRP structure. However, it also contributed to fiber degradation during the winding process and a higher risk of fiber slippage. This degradation was evident as abrasion of the fibers on the composite vessel. The authors of that study investigated only the properties corresponding to the two extreme fiber tension values, without identifying the most efficient one that would retain the beneficial effects of higher tension, such as increased fiber content and strength, while minimizing adverse effects like fiber abrasion. Sinha et al. [16] conducted a comparative study on the influence of fiber tension in filament-wound NOL rings, examining tension levels in 1 kg increments from 1 to 6 kg. Their results showed that specimens achieved maximum hoop tensile strength at a tension of 4 kg, while further increases to 5 and 6 kg resulted in a slight decline in mechanical performance. These findings suggest the existence of an optimal fiber tension, which does not coincide with the highest applied one.

Different mosaic winding patterns influence not only the stiffness and strength of composite structures but also contribute to stress concentrations that can lead to potential failure, including under internal pressurization [17,18]. The mosaic pattern is a parameter often disregarded and challenging to consider during the design process of filament-wound FRP structures, as its influence is still not fully comprehended. Experimental and numerical analyses of various loading conditions have been conducted by researchers in the field. There have been works researching winding pattern influence under different loading cases like axial and radial compression and internal pressure [19,20,21,22,23,24,25]. For instance, Stabla et al. [20] researched radially compressed filament-wound cylinders with different mosaic patterns, finding that the higher amount of interweaving resulted in higher stiffness under radial compression. Loading conditions like axial compression, torsional and internal pressure were analyzed numerically by Menezes et al. [24]. Their key findings indicate that the influence of winding patterns is reduced with the increased number of layers along all investigated loading cases, including internal pressure. This suggests that in thin-walled structures with a small number of layers, the mosaic pattern’s influence is more significant than in thick-walled cylinders, such as high-pressure vessels, where its impact is less pronounced. Rousseau et al. [26] investigated closed-ended pipes subjected to internal pressure with various winding patterns and found that the degree of fiber interweaving correlates with damage propagation, specifically, higher interweaving facilitated more rapid damage growth.

In this study, an internal pressurization method following the recommendations of ASTM C1819-21 [27] using elastomeric inserts was employed to conduct burst pressure tests on filament-wound FRP specimens with varying fiber tensions and mosaic winding patterns. The test setup was designed to simulate hoop stress, which is the most critical stress in pressure pipes and vessels. A similar study was conducted by Madrid et al. [28] where the authors used elastomeric inserts to simulate internal pressure load in cylindrical samples with different winding angles. This method was shown to produce reliable results while avoiding the risks associated with using the hydrostatic burst pressure test. The authors reported this work as the first application of the technique to filament-wound cylindrical structures. Our study aims to further investigate the potential of the elastomeric testing method for filament-wound composite structures. In contrast to earlier work focused on winding angle, our investigation provides a distinct contribution by examining the effects of fiber tension and mosaic winding patterns. Full-field strain analysis was performed using Digital Image Correlation (DIC). An internal structure analysis with Scanning Electron Microscope (SEM) imaging was also performed. This work aims to evaluate the influence of fiber tension and winding pattern on strain distribution and failure modes under internal pressure for thin-walled structures. The novelty of the study lies in the combined analysis of winding tension and mosaic pattern geometry using full-field strain measurements and microstructural observations, enabling a better understanding of their role in achieving an optimized design of filament-wound FRP structures subjected to internal pressure.

## 2. Materials and Methods

### 2.1. Sample Preparation

**Figure 1:**
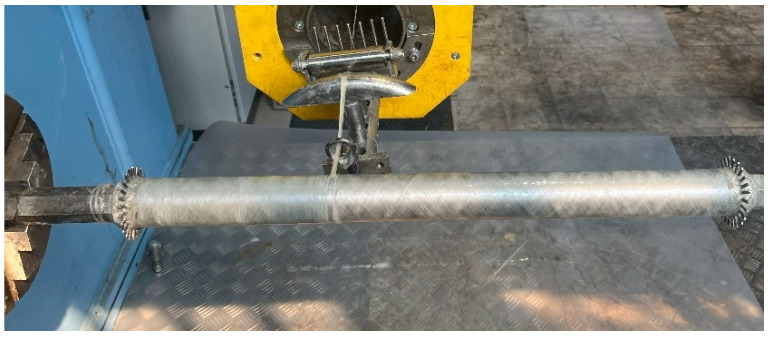
Filament winding of glass fiber/epoxy samples.

The cylindrical samples were prepared on a 4-axis Mikrosam MAW 20 LS4/1 (Prilep, North Macedonia) filament winder using Havel Aeroglass 1200 tex E-glass roving (Svésedlice, Czech Republic) and epoxy resin from Huntsman (Basel, Switzerland)—Araldite LY1564 SP combined with hardener Aradur 3486 BD. A stainless-steel mandrel, 40 mm in diameter and 500 mm in length, was used for the fabrication of all samples. The process of filament winding of the glass fiber/epoxy composite tubes is shown in Figure 1. The mandrel was treated with a release agent before each winding. After winding, the samples were covered with a peel-ply fabric to aid consolidation and allow excess resin and trapped air to escape during the curing process. The samples were then cured in a horizontal oven in a temperature cycle of 1 h at a temperature of 80 °C and 4 h at 120 °C. After curing and cooling to room temperature, the cylinders were removed from the mandrel and cut into samples using a diamond rotary saw. One sample from each set was additionally prepared for DIC analysis.

Two groups of sample sets were prepared: the first group A was used to determine the most efficient fiber tension during winding, while the second group B was designed to evaluate the influence of different mosaic patterns on structural performance during internal pressure loading.

Samples from group A were composed of two layers, each consisting of two plies (+α/−α), the first layer with an angle of ±55° and a mosaic pattern 3/1, while the second was a hoop layer of ±90°. There were 6 sets of specimens with varying tension configurations. The tensions were set apart at the step of 10 N (Table 1).

In the second part of the study, group B of composite samples was produced. All specimens in this group were manufactured using the previously identified most effective fiber tension of 20 N in terms of achieving the highest mechanical properties and structural integrity. The samples differed in the winding pattern, with the following configurations: 2/1, 3/1, 3/3, 4/1. Each configuration was applied to cylindrical specimens made with one ±55° composite layer. The degree of mandrel coverage was controlled to be as close to 100% as practically achievable to ensure that the winding pattern remained the sole variable under investigation. However, producing samples with identical coverage was not feasible, as the coverage inherently depends on the specific geometry of the selected winding pattern.

### 2.2. Application of Fiber Tension

The fiber tension was applied on the creel with a friction brake controlled with a tensioning spring. To accurately capture the resultant tension experienced by the fibers, a force gauge was installed at the end of the wet fiber tow, compensating for frictional losses within the impregnation and feed systems. The tension was measured again after the filament winding process to check for any change coming from the reduced mass of the fiber spool. There was no substantial change to the fiber tension registered after the winding process.

### 2.3. Internal Pressure Testing

The specimens were tested under internal pressure generated using elastomeric inserts. The testing was carried out in accordance with the ASTM C1819−21 [27]. This method was selected due to its suitability for comparative analysis between specimens, as well as its practical advantages over conventional hydraulic burst pressure testing: ease of execution, improved cleanliness, and enhanced safety. Although the standard was originally developed for ceramic matrix composites, it was considered suitable for the present FRP composite tubes. The method provides an approximate representation of internal pressure loading and is therefore particularly appropriate for comparative assessment of specimens tested under identical conditions.

**Figure 2:**
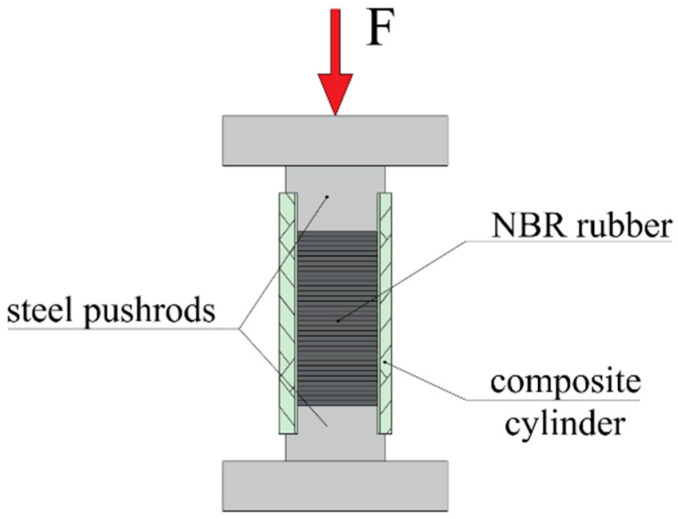
Schematic diagram of the testing setup.

**Figure 3:**
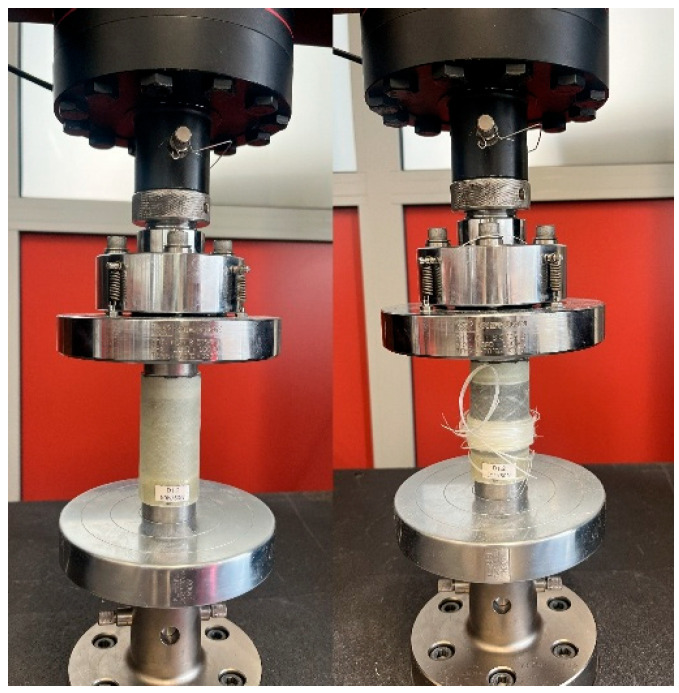
Group A sample before and after pressure testing.

The tests were performed on a 100 kN Instron 5982 universal testing machine (Norwood, MA, USA) equipped for compression testing with additional pushrods to compress the elastomer inside the specimens. The testing setup is schematically presented in Figure 2, while the testing of the sample is shown in Figure 3. The elastomeric inserts were made from NBR (Nitrile Butadiene Rubber) in the form of 2 mm thick, 40 mm diameter discs coated with talc powder to prevent sticking and minimize friction between the discs and the FRP wall. NBR was chosen due to its Poisson’s ratio close to 0.5 [29]. This property ensures that under axial compression the material effectively expands radially, generating uniform internal pressure on the composite tube wall.

The standard defines the minimum required length of the elastomeric inserts and the unpressurized ends as a function of the inner radius (r_i_), wall thickness (t) and Poisson’s ratio (ν) of the composite (1). These requirements are expressed using parameter β, where the length of the elastomeric insert must exceed 9/β, and the unpressurized end sections must be at least 3.5/β.

(1) $$\beta =\sqrt[{4}]{\frac{3(1-\nu^{2})}{{r_{i}}^{2}t^{2}}}$$

For Group A specimens, wall thickness was measured (1.02 mm average), and Poisson’s ratio of the composite was taken as 0.3 [30] for calculation purposes. Based on these parameters, the minimum required length of the elastomeric insert was calculated to be approximately 35 mm, with unpressurized end regions of at least 13 mm. To fully represent the mosaic winding pattern, the pressurized section was extended to 50 mm, and the unpressurized ends were increased to 20 mm. Although Poisson’s ratio was not measured directly, a sensitivity analysis shows that varying ν between 0.1 and 0.5 changes the calculated minimum pressurized length by approximately 2.2 mm. Therefore, the adopted specimen geometry remains safely above the standard requirements. The same measurements were performed for group B specimens, which had a smaller wall thickness due to being made of a single layer. The average thickness of the samples was 0.48 mm. The same specimen geometry was adopted for both groups for consistency, even though the minimum dimensional requirements for group B were lower.

The displacement rate was set to 2 mm/min for group A and 4 mm/min for group B. These values were selected as a compromise: slow enough to allow accurate registration of the deformation process by the DIC system, while also fast enough to minimize the influence of time-dependent effects such as slow crack growth. The displacement rate was chosen according to the stiffness characteristics of each specimen group in order to maintain stable loading conditions and comparable test durations.

### 2.4. Digital Image Correlation

**Figure 4:**
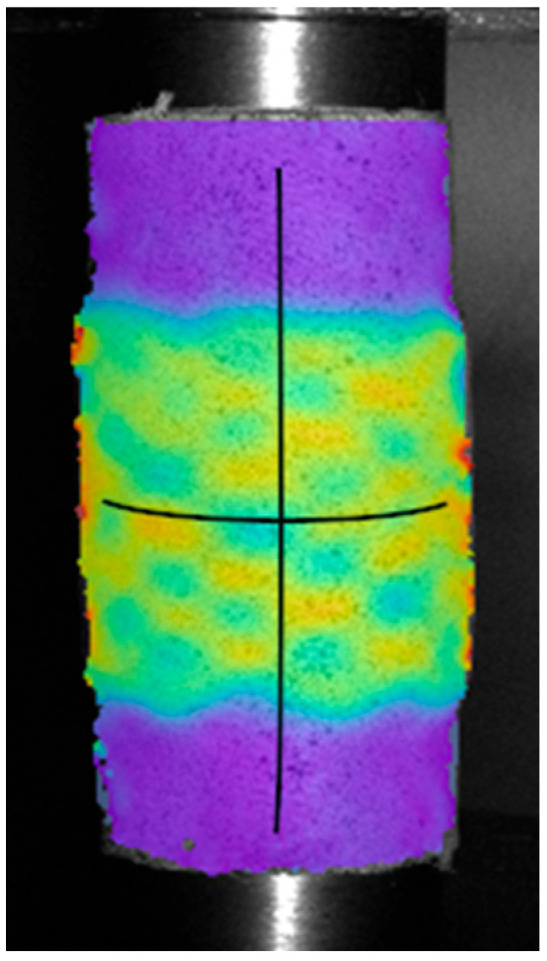
Specimen with probe lines marked.

DIC tracking was used on one prepared sample from each set during the burst pressure test. We acknowledge that analyzing multiple specimens using DIC would improve statistical reliability. However, due to the time-intensive nature of the method, DIC was performed on one representative specimen per configuration. The results are therefore used to provide qualitative and comparative insight into deformation and damage mechanisms rather than statistical evaluation. Prior to DIC measurements, the specimen surfaces were coated with a thin white base layer, followed by the application of a random speckle pattern of black paint using a spray gun to ensure adequate contrast for correlation analysis and strain tracking. The Dantec dynamic Q300 equipment (Skovlunde, Denmark), consisting of two cameras and a red light source, was used along with post-processing software Istra 4D v4.4.6. The sampling frequency was set to 4 Hz. The DIC analysis was performed using a kernel size of 19 × 19 data grid points (ACSP 19 × 19). Quantitative analysis of the strain fields obtained from DIC was performed using virtual line probes. Two lines were defined for each specimen: a circumferential line located at the mid-length of the tube to evaluate hoop strain, and a longitudinal line aligned with the specimen axis to evaluate axial strain (Figure 4). These probe lines enabled the extraction of strain distributions representative of the global deformation state while minimizing the influence of edge effects. The strain values along the defined paths were used to determine characteristic strain levels for each specimen.

### 2.5. Microstructure Imaging

Microstructure imaging was performed using a scanning electron microscope Tescan Vega 3 (Brno, Czech Republic). Samples from Group A were analyzed to evaluate the influence of winding tension on structural integrity. For microstructural evaluation, fragments were cut from tubes that were not subjected to pressure testing and embedded in conductive resin before grinding and polishing. SEM observations were conducted on cross-sections perpendicular to the hoop layer, allowing clear visualization of interlaminar interfaces. Low magnification (200–300×) was used to examine the overall cross-sectional fiber distribution and assess ply integrity, while higher magnification (500–1000×) was applied to evaluate local features, including fiber volume fraction.

## 3. Results and Discussion

### 3.1. Pressure Tests—Group A

**Figure 5:**
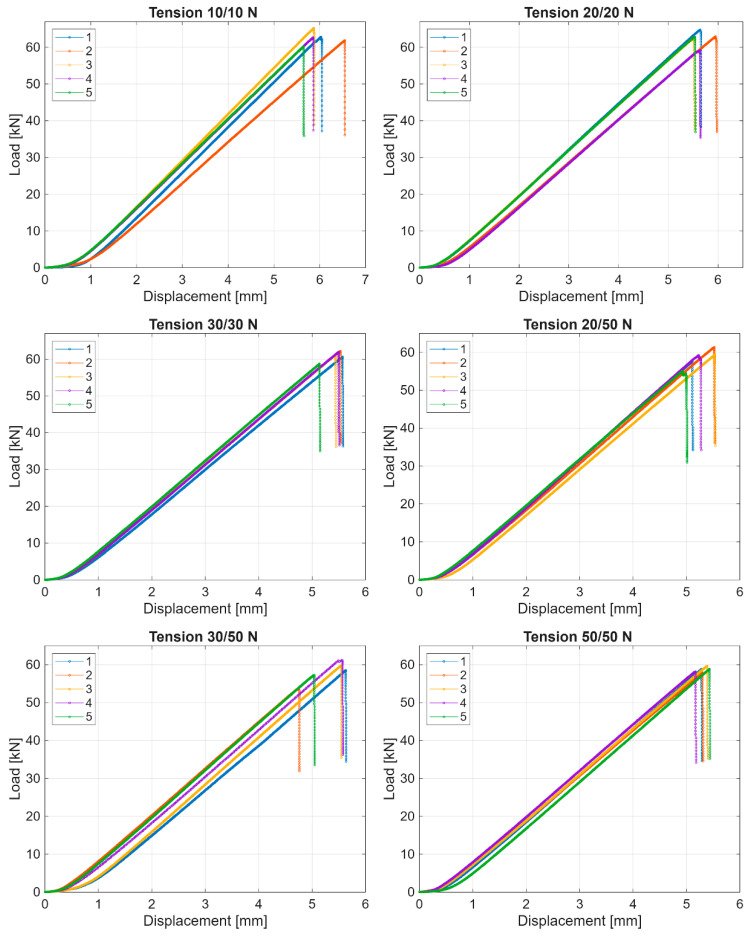
Load–displacement curves for group A samples with different fiber tension values.

The FRP tubes were subjected to internal pressure induced by axial compression of an elastomeric insert, which expanded radially until the specimens reached structural failure. The applied load and the corresponding axial displacement of the testing machine for each specimen set from group A are presented in Figure 5. The compression load–displacement diagrams of all group A sets exhibit a linear response over the entire loading range. The specimens can be classified as linear-elastic-brittle materials. The composite tubes within each series display closely comparable characteristics.

Following completion of the tests, the load–displacement response was converted into internal pressure (*p*) according to Equation (2), where *F* indicates the recorded load and *r_i_* is the inner radius of the tube.

(2) $$p=\frac{F}{\pi \cdot {r_{i}}^{2}}$$

**Figure 6:**
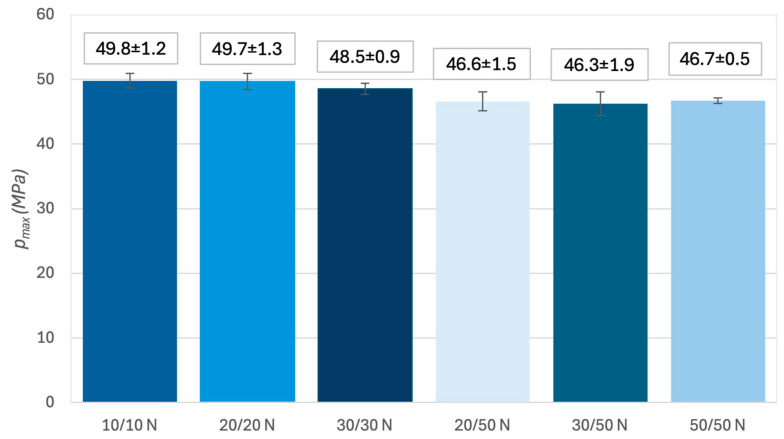
Failure pressure of tubes manufactured with different tension forces.

The failure pressure was identified as the maximum pressure sustained before the loss of structural integrity. The failure pressure calculated across all series remains within a narrow band of 46–50 MPa (Figure 6), showing that while winding tension does influence composite quality, it shows a relatively small impact on pressure failure in thin-walled tubes. While fiber tension is important for ensuring proper fiber alignment and resin consolidation, the failure pressure results suggest that for thin-walled FRP tubes the winding angle [28] and wall thickness dominate the pressure bearing capacity. From a manufacturing standpoint, using lower tension is advantageous when it comes to thin-walled FRP tubes, since it reduces the force required to remove samples from the steel mandrel and lowers the risk of fiber damage. Additionally, lower winding tension (below 50 N) exhibits slightly higher mechanical performance.

### 3.2. DIC Analysis—Group A

**Figure 7:**
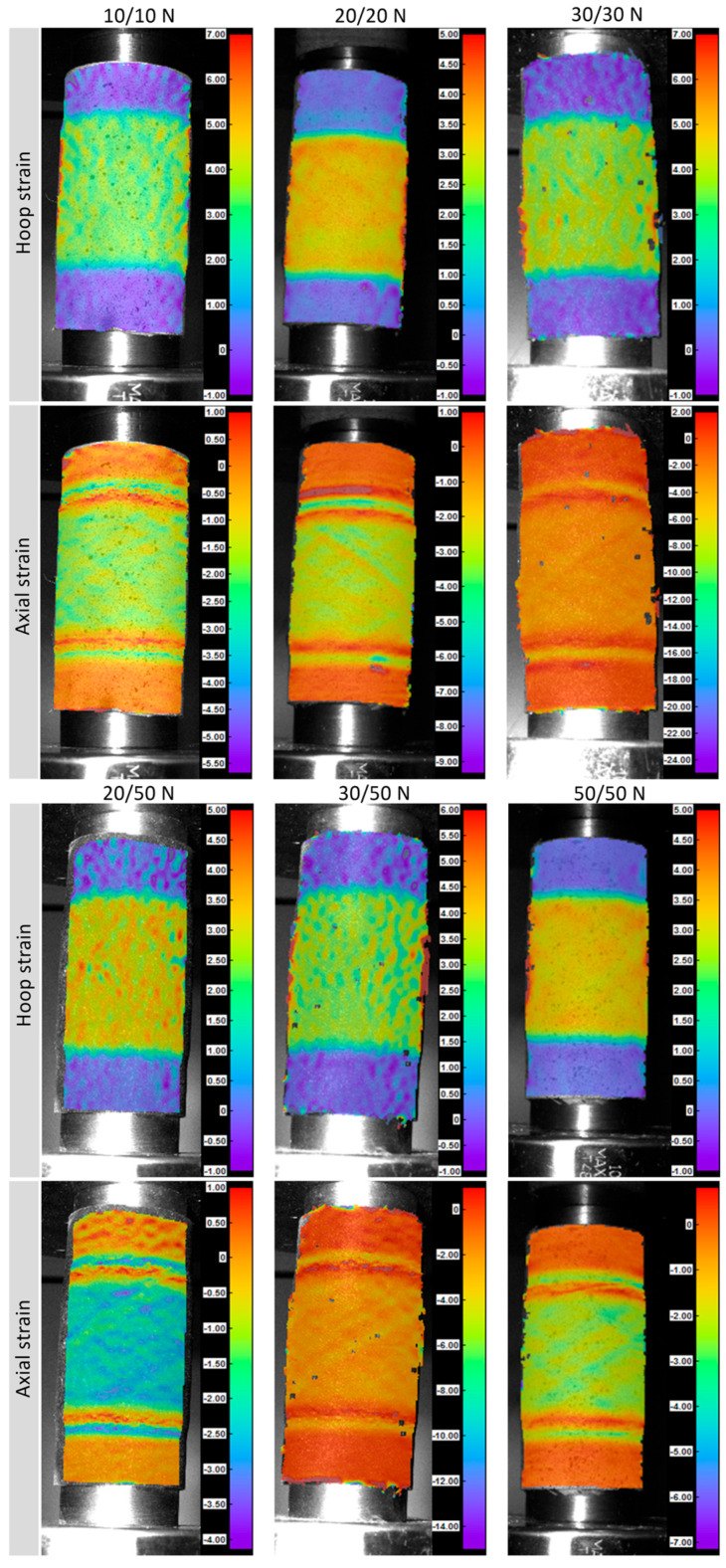
DIC results for group A specimens: hoop strain and axial strain at the maximum applied load.

The DIC results for Group A specimens at the maximum applied load, immediately before rupture, are presented in Figure 7 in the form of axial and hoop strain maps. The hoop strain maps show a mostly uniform strain distribution within the central, loaded region of the specimens, indicating a homogeneous internal pressure state. For visualization purposes, the strain scales were adjusted individually for each specimen to emphasize local strain features. This representation highlights localized variations in the hoop strain field, including strain patterns related to fiber crossover regions, even though the outer layer of the group A specimens is oriented circumferentially. In contrast, the axial strain maps exhibit a non-uniform distribution along the specimen’s length with local tension in the compressed region. It can be explained based on the correlation between the axial and circumferential strain maps. Although the axial strain field is dominated by compressive strains along most of the specimen’s loaded length, localized axial tensile bands are observed. These tensile bands appear at the same axial locations where the circumferential strain starts to arise, indicating the onset of effective radial expansion of the elastomer. In contrast, axial compressive strains are concentrated near the rigid metallic end plates, which do not deform and therefore constrain axial shortening of the tube while delaying radial expansion of the elastomer. As the distance from the end plates increases, this axial constraint is progressively released, allowing the elastomer to expand circumferentially in accordance with the Poisson effect, which in turn induces local axial tensile strains in the tube. This behavior confirms that the observed axial strain heterogeneity is governed by boundary conditions and contact mechanics rather than by material inhomogeneity.

**Figure 8:**
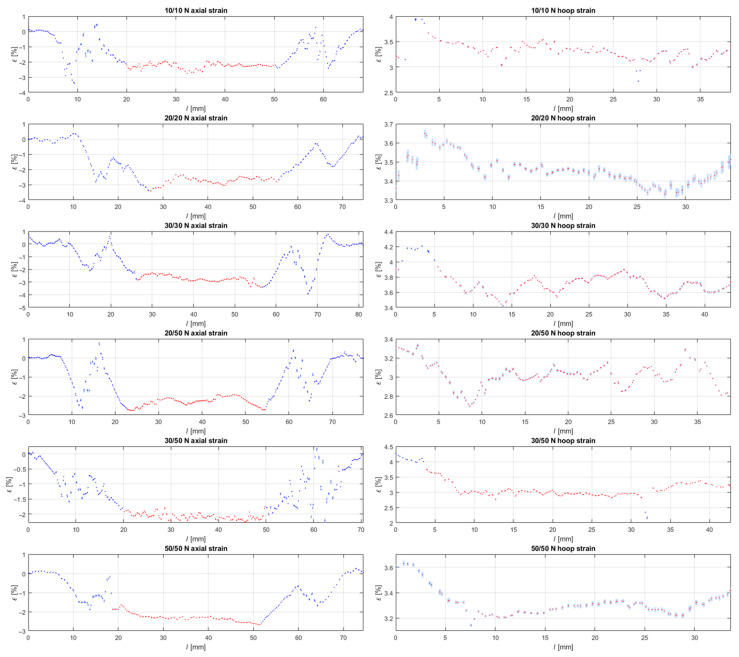
Hoop and axial strain along probe lines for group A specimens (selected data points shown in red).

Quantitative strain data were extracted along two virtual line probes (Figure 4) to evaluate hoop and axial strain. The corresponding strain distributions for each specimen are shown in Figure 8, with individual data points plotted together with their associated deviations. Mean strain values were subsequently calculated based on selected data points extracted from these distributions, which are indicated in red color in the corresponding diagrams. For the axial strain, only data points from the central region of the specimen were considered, where the strain distribution became predominantly uniform. In the case of hoop strain, points that deviated markedly from the remaining data, primarily those located near the specimen edges and affected by higher DIC measurement uncertainty, were excluded from the calculations.

Table 2 summarizes the key experimental results for tested specimens from group A, including the measured wall thickness, failure pressure, mean axial and hoop strains at maximum loading, and the corresponding hoop tensile strength. Hoop tensile strength (*S_hoop_*) can be described by Equation (3), where *p_max_* is the maximum internal pressure, *r_o_* the outer radius of the cylindrical sample, and the *η_m_* is a stress factor. The *η_m_* is a function of radius-thickness ratio and Poisson’s ratio [28,31]. For group A specimens the *η_m_* value was assumed to be 1.08.

(3) $$S_{hoop}={\eta_{m}\cdot p}_{max}\frac{2\cdot {r_{i}}^{2}}{{r_{o}}^{2}-{r_{i}}^{2}}$$

The wall thickness varied between specimen sets, exhibiting a tendency to decrease for samples produced with the highest tension force (50 N), primarily due to resin squeeze-out during manufacturing, while the absolute mass of reinforcing fibers remained constant. Consequently, the calculated hoop tensile strength showed reduced variability among the different specimen sets, except for the 20 N samples, for which the wall thickness was comparable to that of the 50 N specimens. One-way ANOVA revealed significant differences in hoop tensile strength among the analyzed configurations (*p* < 0.001), confirming that the winding tension has a measurable effect on the mechanical response of composite tubes. However, post hoc Tukey tests indicated that only the 10/10 N and 20/20 N configurations differed significantly from the remaining groups, with no statistically significant differences observed among the other configurations (*p* > 0.05). This statistical analysis supports the observation that, despite measurable differences, configurations with a hoop-layer wound with higher tension (50 N) exhibit comparable pressure performance and hoop tensile strength. This observation suggests that, within the investigated range, the winding tension applied to the hoop layer plays a dominant role in determining the pressure at failure. Since internal pressure primarily generates circumferential (hoop) stresses in thin-walled cylindrical structures, the mechanical response at failure is governed predominantly by the properties and consolidation state of the hoop-oriented plies. Variations in the tension applied to the ±55° layers appear to have a comparatively minor influence on the pressure-driven failure mechanism.

A similar trend has been reported in Ref. [16], where, despite the application of higher winding tension (up to 6 kg/tow), the optimal mechanical performance was achieved at approximately 4 kg/tow, indicating that increasing tension beyond a certain level does not lead to further improvement. All specimens exhibited abrupt failure governed by a hoop-dominated brittle fracture mechanism, manifested by longitudinal splitting of the tube wall and fiber breakage aligned with the circumferential stress direction. Overall, the findings suggest that lower winding tensions can be employed without compromising pressure performance, while offering potential advantages in manufacturing.

### 3.3. Microstructure Analysis—Group A

**Figure 9:**
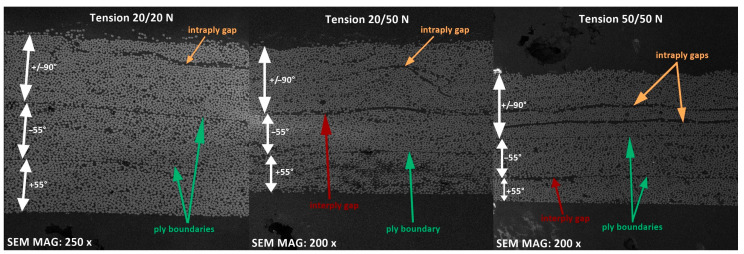
SEM imaging of the samples manufactured with varying tension forces.

SEM analysis was performed on three selected configurations: 20/20 N, 20/50 N, and 50/50 N (Figure 9). These variants were chosen to represent distinct manufacturing conditions and corresponding mechanical responses, with the 20/20 N specimens exhibiting higher failure pressure (49.7 MPa), while the 20/50 N and 50/50 N specimens showed lower and nearly identical failure pressures (46.6 MPa and 46.7 MPa, respectively). For microstructural evaluation, fragments were cut from tubes that were not subjected to pressure testing. SEM observations were conducted on cross-sections perpendicular to the hoop, allowing clear visualization of interlaminar interfaces. Although the measured wall thicknesses of all configurations were comparable, apparent variations in thickness are visible in the micrographs. This is due to the fact that the analyzed sections were taken from curved tube fragments (arc segments), which made it challenging to position all samples identically during inclusion. Furthermore, the grinding and polishing procedure slightly reduced the dimensions of the included fragments and modified the observed surface geometry.

Although fiber volume fraction was evaluated from SEM images, its quantitative assessment proved challenging due to significant local variability within individual specimens, with higher values observed in well-consolidated regions and lower values in areas with reduced interlaminar integrity. Therefore, the analysis focused primarily on interlaminar bonding and overall ply integration, assessed using lower magnification imaging, rather than on detailed high-magnification evaluation of local fiber content. The 20/20 N specimens exhibited well-integrated interfaces between successive plies (+55°/−55°/+90°/−90°), with minimal visible voids. In contrast, the 20/50 N and 50/50 N specimens showed the presence of interply gaps, indicating reduced consolidation between layers. Additionally, specimens manufactured with a winding tension of 50 N exhibit a higher occurrence of intraply gaps in the hoop layer. These microstructural differences suggest that variations in winding tension distribution affect interlaminar compaction and bonding quality, which may contribute to the observed differences in failure pressure.

This observation is consistent with previous studies, which identified fiber tension as a key parameter influencing porosity, fiber alignment, and structural integrity. M. Yadav et al. [32] showed that increasing winding tension enhances fiber bed compaction and resin flow, reducing void content. In the study the best mechanical performance of flat wound samples was achieved at a tension of 63 N. However, similarly as in this study excessive tension can lead to adverse effects, including voids and delamination. Different values of the most efficient winding tension level in this study and Ref. [32] highlights that the winding tension is not universal and depends strongly on the structural geometry and loading conditions.

### 3.4. Pressure Tests—Group B

**Figure 10:**
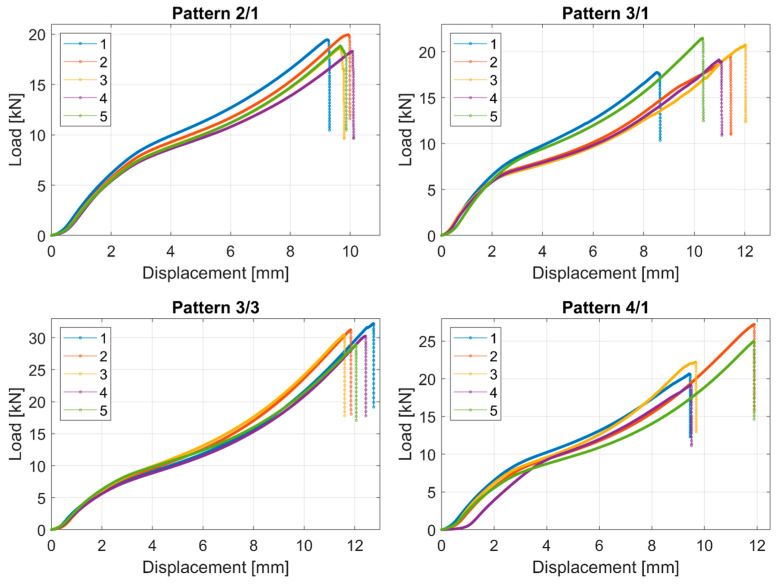
Load–displacement curve for patterns 2/1, 3/1, 3/3, 4/1 (group B).

Figure 10 presents the load–displacement curves for each specimen from group B. Slight separations between the curves are observed in the 3/1 and 4/1 patterns; however, no clear correlation explaining this behavior is evident. This variability is likely related to the specific region from which the specimen was cut. It can be noticed that each curve begins with a nonlinear part, which is caused by the initial deformation and compression of the rubber discs without the cylinder yet deforming. The rubber then starts to transmit pressure onto the composite walls. Then the sample deforms in a quasi-linear manner up to reaching the burst pressure when the sudden drop in load is registered. The quasi-linear deformation is caused by the fibers realigning and a slight change of the winding angle caused by the expansion of the sample under internal pressure. The final stage of loading proceeds in a linear manner, indicating that the fiber orientation has stabilized, and the applied load is carried through the elastic deformation of the fibers.

**Figure 11:**
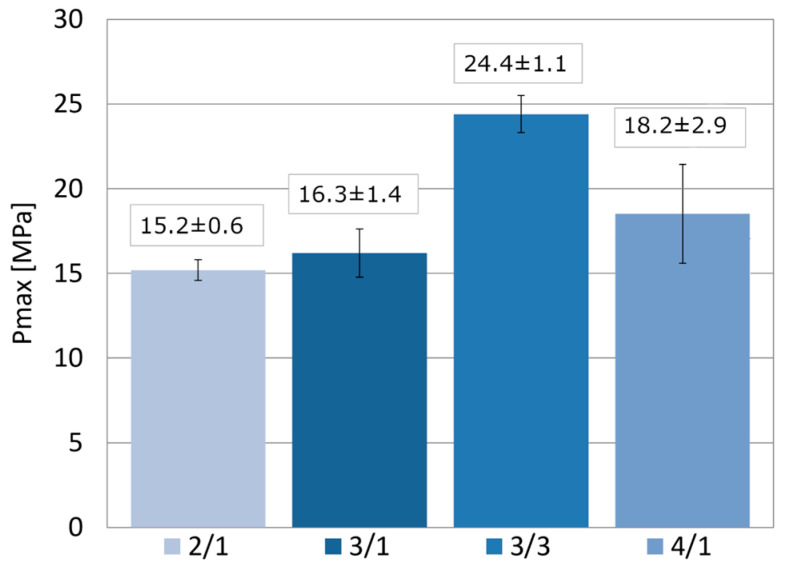
Failure pressure of tubes with different winding patterns.

Results for group B specimens are presented in Figure 11. The failure pressure for each sample set has been calculated based on the maximum force registered by the machine (Equation (2)).

**Figure 12:**
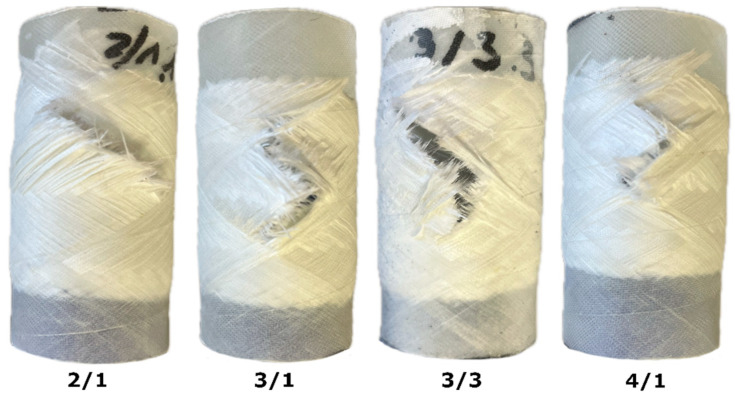
Specimens (group B) after burst test.

Representative specimens from each set after the burst pressure test are shown in Figure 12. In all configurations, failure propagated predominantly along the pattern repetition boundaries of the diamond-shaped mosaic pattern, indicating that these regions act as preferred crack paths. The fracture surfaces closely follow fibers located at the repeating edges of the diamond pattern, suggesting that local stress concentrations associated with the winding pattern govern crack initiation and propagation. While all specimens exhibit a broadly similar failure mode, the 3/3 configuration shows a more distributed damage zone with less clearly defined crack propagation paths. This behavior can be attributed to the increased density of crossover regions. The more complex pattern, lacking distinct diamond boundaries, reduces the tendency for crack growth to follow a single preferential direction, thereby increasing the energy required for damage propagation. At each crossover region, the local change in fiber orientation in the ply alters the stress field, forcing the crack to repeatedly change its propagation direction, resulting in a more complex fracture path. This contrasts with the findings of Rousseau et al. [26], who reported that a higher degree of interweaving promoted more rapid damage propagation. This behavior indicates a different stress redistribution mechanism, consistent with the higher deformation capacity and burst resistance observed in this configuration.

Similar trends were reported by Stabla et al. [20], who observed that an increased degree of interweaving enhances the strength of composite structures under radial compression. Although it is a different type of loading, similar mechanisms of crack path formation may be present. In particular, the more frequent interweaving of the fibers can act as a barrier and block the crack growth, requiring more energy to propagate. In contrast, simpler winding patterns like 2/1, with fewer crossover areas, allow cracks to develop more easily along straight paths.

### 3.5. Digital Image Correlation Analysis—Group B

**Figure 13:**
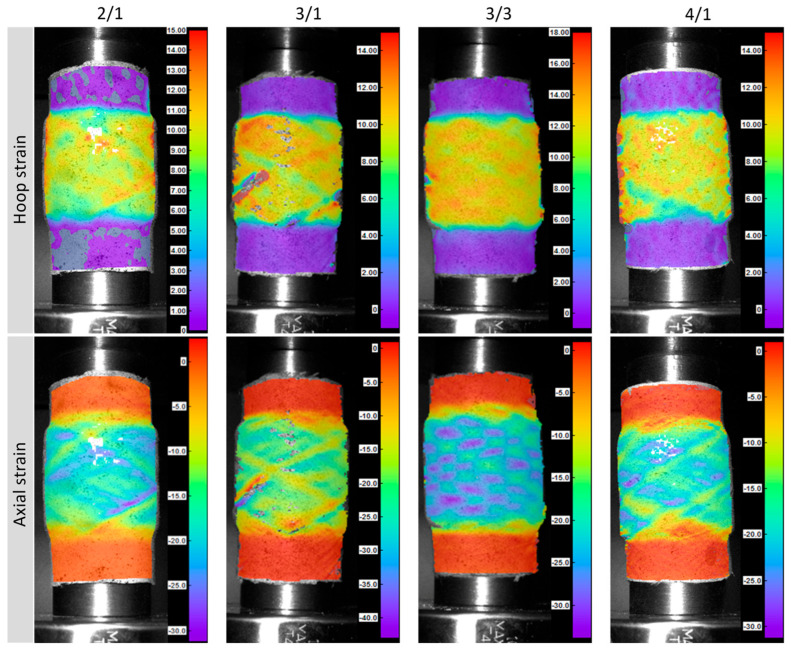
DIC results for group B specimens: hoop strain and axial strain at the maximum applied load.

DIC analysis results for the group B specimens are presented in Figure 13. The images show axial and hoop strain maps immediately before burst for all four tested winding patterns. The strain scales were adjusted individually for each specimen to highlight local strain features. In the pressurized region of each sample, distinct line patterns corresponding to the winding pattern are clearly visible, in both axial and radial strain maps, with the highest strain concentrations occurring at fiber crossover regions. For specimen 3/1 a localized high-strain zone is observed along a diamond-shaped region of the mosaic pattern, reaching strain values of approximately 14%. This region coincides with the burst location. The axial strain maps exhibit peak values in the same crossover regions as the hoop strain, which confirms that the crossover zones play a critical role at the initiation of the failure.

**Figure 14:**
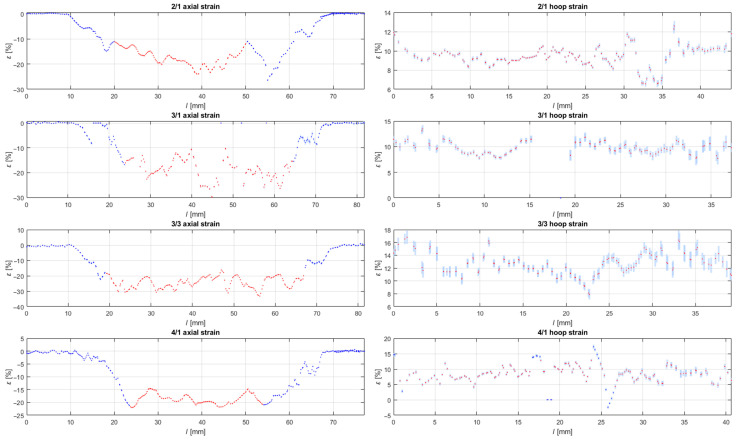
Hoop and axial strain along probe lines for group B specimens (selected data points shown in red).

Quantitative strain data were extracted, processed, and presented using the same virtual probe line procedure described for group A. The strain distributions and corresponding mean values shown in Figure 14 were obtained following an identical data treatment approach.

The graphs presented in Figure 14 show strains to be visibly more irregular compared to group A specimens. This is caused by the winding pattern related to the ±55° winding angle and the inhomogeneity of the material along the probe line. Distinct variations are associated with fiber crossover regions, where local strain concentrations occur.

Table 3 presents parameters of the group B specimens. For group B the *η_m_* value was assumed to be 1.05. There are noticeable differences in mechanical performance between the tested winding patterns. The parameter used for comparison between the samples is the hoop tensile strength, as it accounts for both the failure pressure and the specimen thickness. One-way ANOVA revealed significant differences in hoop tensile strength among the configurations (*p* < 0.001). It confirms that the change in winding pattern has a measurable influence on the strength of the composite tubes. Post hoc Tukey tests showed that the 3/3 pattern differed significantly from all other configurations, while no significant differences were found between the 2/1, 3/1, and 4/1 patterns (*p* > 0.05). The 3/3 configuration stands out, exhibiting substantially higher hoop tensile strength than all other specimens, together with the largest axial and hoop strains at failure. This indicates a greater ability to accommodate deformation and redistribute stress before bursting. In contrast, the 2/1, 3/1, and 4/1 patterns show relatively similar hoop tensile strengths and strain-at-burst values, suggesting less efficient load transfer compared to the 3/3 specimens. Overall, the results confirm that variations in winding pattern and crossover numbers lead to pronounced differences in structural response, overshadowing the influence of geometric parameters such as wall thickness.

Despite the valuable insights provided by this study, several limitations should be acknowledged. The number of specimens tested was limited to five, which may affect the statistical generalization of the results. In addition, the manufacturing process introduces variability of the composite structure, including randomly occurring defects, which may influence the structural response of individual specimens. The DIC analysis was conducted on one representative specimen per configuration and therefore, cannot serve as a statistical analysis, only as a qualitative assessment of strain distribution and damage mechanisms. Furthermore, the experiments were performed under quasi-static internal pressure loading using a specific testing setup, which may limit the direct transferability of the results to other loading conditions or structural configurations. However, it remains suitable for comparative analysis within the scope of this study.

## 4. Conclusions

The present study investigated the influence of filament winding tension and mosaic pattern on the mechanical performance and microstructure of thin-walled FRP tubes subjected to internal pressure generated by axial compression of an elastomeric insert. Based on the experimental results, the following conclusions can be drawn:

1. The failure pressures of the group A specimens were confined to a relatively narrow range (approximately 46–50 MPa), indicating that within the investigated tension range, winding tension has a limited effect on the pressure-bearing capacity of thin-walled structures.
2. ANOVA analysis revealed significant differences in hoop tensile strength among all investigated configurations. Post hoc Tukey testing showed that only the 10/10 N and 20/20 N configurations differed significantly, while no statistically significant differences were found among the remaining configurations. In particular, configurations manufactured with a hoop-layer winding tension of 50 N (20/50 N, 30/50 N, and 50/50 N) exhibited nearly identical failure pressure and hoop tensile strength, confirming that the mechanical response under internal pressure is governed predominantly by the circumferential (hoop) layers.
3. All group A specimens failed abruptly by a hoop-dominated brittle fracture mechanism, characterized by longitudinal splitting and fiber breakage aligned with the circumferential stress direction. This behavior is attributed to the dominance of circumferential stress under internal pressure, which leads to dominant loading and failure of hoop-oriented fibers. The limited tensile strength of the fibers promotes rapid crack propagation, resulting in abrupt longitudinal splitting.
4. SEM analysis showed that specimens manufactured with lower and uniform winding tension (20/20 N) exhibited improved interlaminar consolidation, whereas configurations incorporating higher hoop-layer tension displayed visible interply gaps. These microstructural differences suggest that winding tension influences interlaminar bonding quality. Although increased winding tension is considered beneficial for fiber alignment and laminate compaction, the present results indicate that, for thin-walled tubes subjected to internal pressure, lower and more uniform tension levels provide slightly superior pressure performance while promoting better interlaminar integrity.
5. Damage of group B specimens did not propagate strictly along crossover regions but instead followed paths influenced by the mosaic pattern geometry. The increased number of crossover regions in the 3/3 pattern contributed to the higher mechanical strength of the specimen under internal pressure loading. The resulting damage propagation paths were more complex than in simpler winding patterns, preventing crack growth along a single dominant trajectory. This distributed damage evolution promotes more effective stress redistribution, which contributes to the improved burst resistance of the structure.
6. Strain fields in helical layers are inherently heterogeneous and strongly dependent on the winding pattern. This heterogeneity leads to localized strain concentrations that govern where deformation accumulates and failure initiates. Consequently, the winding pattern plays a decisive role in controlling the mechanical response under pressure loading.

## Figures and Tables

**Table 1:** Fiber tension value configurations of filament-wound FRP tubes (group A).

Set	Fiber Tension	
	Helical Layer	Hoop Layer
10/10	10 N	10 N
20/20	20 N	20 N
30/30	30 N	30 N
20/50	20 N	50 N
30/50	30 N	50 N
50/50	50 N	50 N

**Table 2:** Geometric and mechanical parameters of group A specimens.

Tension	Failure Pressure (MPa)	Wall Thickness (mm)	Axial Strain Failure (%)	Hoop Strain Failure (%)	Hoop Tensile Strength (MPa)
10/10 N	49.8 ± 1.2	1.028 ± 0.037	2.27 ± 0.19	3.30 ± 0.14	946 ± 37
20/20 N	49.7 ± 1.3	0.976 ± 0.015	2.74 ± 0.24	3.45 ± 0.07	1074 ± 38
30/30 N	48.5 ± 0.9	1.028 ± 0.037	2.74 ± 0.26	3.70 ± 0.11	995 ± 44
20/50 N	46.6 ± 1.5	1.001 ± 0.062	2.31 ± 0.23	3.02 ± 0.14	984 ± 73
30/50 N	46.3 ± 1.9	0.990 ± 0.032	2.08 ± 0.11	3.07 ± 0.21	985 ± 42
50/50 N	46.7 ± 0.5	1.007 ± 0.016	2.30 ± 0.22	3.29 ± 0.06	978 ± 20

**Table 3:** Geometric and mechanical parameters of group B specimens.

Winding Pattern	Failure Pressure (MPa)	Wall Thickness (mm)	Axial Strain Failure (%)	Hoop Strain Failure (%)	Hoop Tensile Strength (MPa)
2/1	15.2 ± 0.6	0.401 ± 0.035	17.5 ± 3.4	9.4 ± 1.1	792 + 60
3/1	16.3 ± 1.4	0.470 ± 0.060	19.0 ± 4.4	9.8 ± 1.2	722 + 73
3/3	24.4 ± 1.1	0.490 ± 0.019	24.4 ± 3.7	12.6 ± 1.8	1088 + 43
4/1	18.2 ± 2.9	0.472 ± 0.032	18.9 ± 2.0	8.5 ± 2.0	798 + 86

## Data Availability

The original contributions presented in this study are included in the article. Further inquiries can be directed to the corresponding author.

## Abbreviations

The following abbreviations are used in this manuscript:

DIC	Digital Image Correlation
SEM	Scanning Electron Microscopy
NBR	Nitrile Butadiene Rubber
FRP	Fiber-Reinforced Polymer
